# The Psychometric Properties of the Gilliam Autism Rating Scale (GARS-3) with Kurdish Samples of Children with Developmental Disabilities

**DOI:** 10.3390/children9030434

**Published:** 2022-03-19

**Authors:** Sayyed Ali Samadi, Hana Noori, Amir Abdullah, Lizan Ahmed, Barez Abdalla, Cemal A. Biçak, Roy McConkey

**Affiliations:** 1 Institute of Nursing and Health Research, Ulster University, Newtownabbey BT37 0QB, Northern Ireland, UK; r.mcconkey@ulster.ac.uk; 2 Northern Ireland and Bahoz Centre for Children with Developmental Disabilities, Erbil 44002, The Kurdistan Region of Iraq, Iraq; 3 Evaluation Unit, Bahoz Centre for Children with Developmental Disabilities, Erbil 44002, The Kurdistan Region of Iraq, Iraq; hanan@bahozcenter.com (H.N.); amir@bahozcenter.com (A.A.); lezan@bahozcenter.com (L.A.); barez@bahozcenter.com (B.A.); cemal.a.bicak@bahozcenter.com (C.A.B.)

**Keywords:** autism, autism spectrum disorder, GARS, assessment, psychometrics, developmental disabilities, low and middle-income countries, Kurdistan

## Abstract

There is marked variation internationally in the prevalence of children identified as having autism spectrum disorders (ASD). In part, this may reflect a shortage of screening tools for the early identification of children with ASD in many countries. This study aimed to evaluate the Kurdish translation of the Gilliam autism rating scale—third edition (GARS-3), a scale commonly used in Western countries that evaluates six domains related to the ASD definition from the Diagnostic and Statistical Manual of Mental Disorders (DSM) 5, notably Restricted/Repetitive Behavior, deficits in Social interaction and Social Communication, as well as differences in Cognitive Style, Maladaptive Speech, and Emotional Response. GARS-3 assessments were completed through interviews with parents of 735 children, 442 (53%) of whom were diagnosed with ASD. 165 (22%) with an intellectual disability, 49 (7%) with communication disorders, and 133 (18%) typically developing children. The reliability, construct, and the predictive validity of the scale was assessed, and the scores suggestive of a child having ASD were identified. The factor structure was broadly replicated, especially on items relating to social interaction and social communication. The cutoffs for the total scores that were indicative of possible ASD had a high degree of specificity and sensitivity in distinguishing children with ASD from typically developing peers. Some children with I.D. and communication disorders may also score above the threshold, and further assessments should be sought to confirm the presence of autistic traits. Although GARS-3 could be recommended for use in Kurdistan and possibly similar cultures, further prospective research is needed to confirm a diagnosis of assessment with children who score above and below the cutoff scores identified in this study. Moreover, the development of normative data drawn from Kurdish samples of children would be advantageous, although ambitious, given the lack of diagnostic services in many low- and middle-income countries.

## 1. Introduction

Although the prevalence of children with autism spectrum disorders (ASD) is increasing internationally, there are marked variations in the rates reported, with those in affluent western countries far surpassing those in low and middle-income countries [1]. This is partly due to the disparities in access to assessment and diagnostic services. This is reflected in the lack of trained professionals that are able to undertake the assessment and diagnosis of ASD (and indeed other developmental disabilities) but also a dearth of culturally validated tools to use to reliably identify children with ASD [2] and especially at a young age when early intervention can be implemented and their families supported [3].

A starting point in addressing the challenge of finding suitable assessment instruments is to explore the transfer of tools of proven effectiveness from Western countries to other cultures. Moreover, if these tools are designed to be used by community personnel rather than specialists, then they will be more readily implemented in low and middle-income countries [4]. This approach was successfully adopted in Iran using the Gilliam autism rating scale (GARS)—second edition) [5] that was widely used in educational and clinical settings across the U.S.A. This scale comprises easily understood items and is designed to provoke information from caregivers and people such as parents and teachers who know the child to judge the individual ability in each item.

The Iranian version of GARS-2 was tested with parents of 658 children [5]. Of these, 442 had a confirmed diagnosis of ASD provided by clinicians in the special education organisation or social welfare organisation; 112 intellectually disabled children; and 102 typically developing individuals. Across these three groups, the psychometric properties of the GARS-2 were broadly replicated, and children with AS were clearly distinguished from the other two groups based on the cut-offs that maximized the scale’s sensitivity and specificity.

Since then a revised version of this scale (GARS-3) has been developed that overcame some of the shortcomings of this tool, notably an increased number of items, particularly relating to language and communication [6]. The revised version also incorporates diagnostic domains and criteria changes as proposed in DSM-5 [7]. In the United States, a more thorough psychometric assessment of the revised GARS has been undertaken with nearly 2000 children who had a confirmed diagnosis of ASD [8]. Thus far, however, it does not seem to have been used in less affluent countries. In this instance, the opportunity arose to access a sample of Kurdish children and families from northern Iraq. However, this culture is represented in many neighboring countries and immigrant communities internationally.

The present study had two main aims:

1. To replicate the process for the translation and psychometric evaluation of autism spectrum assessment tools as had been developed in Iran [5] and in Turkey [9].
2. To identify the properties of the Kurdish version of GARS-3.

## 2. Method

### 2.1. The Kurdistan Context

The study was located in the semi-independent Kurdistan region of northern Iraq. In the absence of a formal national census, the exact population and demographics of the Kurdistan region are uncertain, but in 2014, the population was estimated to be 5.1 million in nearly 1 million households [10]. An estimated 35% of the population was younger than 15 years and 13% of the households had a member with an intellectual or physical disability. There are no state-provided education, health or social services for children with developmental disabilities, and families rely mainly on private services that require fees. Currently, the Kurdistan Region is hosting around 1.2 million refugees who have been displaced after the conflicts inside Iraq and in neighboring Syria [11].

### 2.2. Description of GARS-3

The GARS-3 is designed to screen for ASD in individuals between the ages of three and 22 and can be administered to both verbal and non-verbal individuals [12]. The scale consists of 58 items grouped into Restricted/Repetitive Behaviors (13 items), Social Interaction (14 items), Social Communication (nine items) and Emotional Responses (eight items). For children with verbal communication two further sets of items are used: Cognitive Style (seven items), and Maladaptive Speech (seven items).

The child’s present behaviours are scored on a Likert scale consisting of four-points from ‘Not at all like the individual’ (0), ‘Not much like the individual’ (1), ‘Somewhat like the individual’ (2), ‘Very much like the individual’ (3). If there is uncertainty about rating an item, the child should be observed for longer, or information can be obtained from previous observations.

The ratings can be given by parents alone or more preferably through an interview with an assessor and with opportunities to observe the child.

The scores of each subscale are added up and a standardised score is derived based on normative data from American children with ASD Higher scaled scores on a subscale represent increasingly severe autistic behavior. The scaled scores can be totaled to calculate a composite score known as the Autism Index, which is considered to be the best and most reliable standardised score for identifying an individual with ASD The children’s Index scores are grouped according to the severity level of their autism (unlikely; probable; very likely) along with an indication of their likely support needs (No ASD support required; Minimal support required; Substantial support required; Very substantial support required). These categorizations reflect the diagnostic criteria used in DSM-5.

Reliability and validity data for the English version of GARS-3 is available based on a normative sample of 1,859 individuals; aged 3 to 22 years from 48 states in the U.S.A. with a diagnosis of ASD (61% of whom had only ASD and 39% had AS with other co-morbidities.) [8]. Average Cronbach’s alphas of 0.94 and 0.93 were recorded for the Autism Index 4 and Autism Index 6, based respectively on four or six subscales. Test–retest reliabilities (based on 122 individuals who were rated twice within in a 2-week period) ranged from 0.76 to 0.87 on the subscales and were 0.90 for both the Autism Index 4 and Autism Index 6.

An exploratory factor analysis confirmed the construct validity of the scale in relation to DSM-5 diagnostic indicators [8]. Overall, the items accounted for 46% of the total variance with the first contributing the highest proportion, namely items relating to impairments in social interaction. The second factor reflected items related to repetitive, stereotyped behaviors and the third factor was composed of the items that measure social communication. Three further factors were identified as noted above which accounted for decreasing amounts of variance overall.

In order to assess the scale’s predictive validity, the sensitivity and specificity of the autism index scores in distinguishing children with ASD from typically developing children were reported to be: sensitivity = 0.96 (4 subscales) 0.95 (6 subscales), specificity = 0.95 (4 subscales) 0.97 (6 subscales). However, the index had lower specificity scores when comparisons were made between children with ASD compared to children with other developmental disabilities (specificity 0.78 (4 subscales) 0.84 (6 subscales)).

### 2.3. Procedures

The first author translated the GARS-3 into Kurdish, considering the usual safeguards of back-translating. The translated Kurdish version was reviewed for language clarity and appropriateness in the Kurdish culture at the first stage of the procedure by the co-authors who were experienced psychologists and therapists at the Bahoz Center for Children with Developmental Disabilities in Erbil city.

The translated items were then pilot tested with 22 Kurdish families from different socioeconomic backgrounds whose children had been referred for a diagnostic assessment. Based on feedback from caregivers, eight of the 58 items were reworded to improve their clarity and relevance to Kurdish culture.

Through an announcement posted on the social media pages of the Bahoz center, parents and caregivers were informed about the study’s aims. The assessors presented information about the project orally before their interview. Before the interview, consent to participate was obtained from all of the participants. Most caregivers were seen individually in the Bahoz center at the clinics they were attending, but others were seen at home, particularly those who had typically developing children or those with I.D. who lived in cities other than Erbil.

For all participants, the scale was used in a structured interview format with the child present(s). Eight practitioners undertook these with a background in psychology (5 clinical psychology, two special education and one educational psychology) who had over two years of experience with children with ASD They were trained by the first author (a certified ADOS and ADI-R trainer) in a three-day workshop that covered ASD signs and symptoms, ASD criteria in DSM-5, and the administration of GARS-3. The assessors submitted video recordings of interviews undertaken with at least two caregivers of individuals with ASD to improve the level of consistency in administering the scale between the different assessors.

### 2.4. Participants

All subjects gave their informed consent for inclusion before they participated in the study. The study was conducted in accordance with the Declaration of Helsinki, and the protocol was approved by the Ethics Committee of the Bahoz Center for children with developmental disabilities (Project identification code = BCRD12-2020 approved in 14 September 2020). In the absence of a clear national protocol, this committee adheres to the seventh revised version W.M.A. of the Helsinki Declaration on Medical Research involving Human Subjects issued on 19 October 2013. The parents/caregivers were informed about the study and those who agreed to participate and provided written consent were included.

Four groups of children and parents were recruited, for a total of 735 participants in all.

Children with ASD (*n* = 388): Parents of children with ASD and other developmental disabilities were recruited from special centers and some from parental groups of children with developmental disabilities. These children had a diagnosis of ASD based on D.S.M. 5 criteria by clinicians at the Bahoz Center or a previous diagnosis by medical staff such as psychiatrist or pediatrician retrieved from the medical records presented to the Bahoz Centre.

Children with an intellectual disability (ID) (*n* = 165). In the absence of the standardized I.Q. test to confirm the intellectual disabilities, the ID group were mainly individuals with Down Syndrome and other conditions that are associated with ID or had this diagnosis from the pediatricians based on their clinical presentation and developmental assessments.

Children with communication disorders (CD) (*n* = 49): These children were identified through multi-disciplinary assessments undertaken by clinicians at the Bahoz Center and based on the DSM-5 criteria of Social (Pragmatic) Communication Disorder.

The typically developing (TD) sample (*n* = 133) was recruited from children’s clinics, schools, and volunteer groups that the administrators had informed by social media and through their network of acquaintances.

Overall, the children’s age range was from 3 to 18 years, with a mean of 6.4 years (SD 3.2). Many more boys were identified compared with girls, as is commonly the case with ASD (78% vs. 22%). The gender ratio for the children with ID was (64% male vs. 36% female), communication disorders (76% male vs. 24% female) and typically developing (51% male vs. 49% female). In all, 58% of the children used verbal communication, but 42% were non-verbal. The latter proportion was the highest in children with AS (57%), followed by children with ID (38%) and 5% of typically developing children.

The children were recruited mainly from the city of Erbil (83%) or the neighboring residential areas (11%), with the remainder of the Kurdish individuals from outside the Kurdistan region of Iraq (6%).

Both parents were the primary respondents for 47% of the children, mothers for a further 35% of children, fathers only for 11% of the children, and other family members of 7% of the children.

### 2.5. Inter-Rater Study

To establish the test-retest reliability of the scales, the GARS was first administered to the caregivers of 39 children (age mean was 10.4 years in a range from six to 21 years) who had been diagnosed with ASD by various clinicians and were referred to the Bahoz Center for training and rehabilitation services or further assessment. Two weeks later, caregivers were requested to complete the scale for a second time without reference to their previous rating.

### 2.6. Data Recording and Analysis

The ratings given to each child were recorded on paper and were later transferred to spreadsheets for data analysis. A sample of records were cross-checked by the first author and data cleaning procedures were undertaken prior to statistical analysis. The latter was conducted in SPSS version 27 (SPSS Inc., Chicago, IL, USA)using a range of analyses described in the results section.

## 3. Results

The caregivers reported few difficulties in understanding and responding to the items during the interviews. On average, each session took around 45 min to complete all the items, but extra time was spent debriefing parents. Many caregivers spoke with gratitude for the opportunity to discuss their children with the assessors.

### 3.1. Factor Analysis

Factor analyses were first undertaken to determine the replication of the six factors represented in GARS-3. The data was suitable for these analyses as the K.M.O. and Bartlett’s Test confirmed (approximate chi square = 14,586.7; *p* < 0.001).

First, a factor analysis was undertaken of the items in the four subscales on which all children were rated (*n* = 735). A four factor solution using a varimax rotation accounted for 46% of the variance (as in the GARS-3 standardisation sample). The first factor largely replicated the Social Interaction items of GARS-3, with 12 of the 13 items loading on this factor plus one behaviour item (with loadings of 0.55 or higher). This factor accounted for 17.6% of the total variance. The second factor included all nine of the Social Communication items (with loadings of 0.5 or greater) and accounted for 13% of the variance. The third factor consisted largely of Behavioral items—eight in all from 13 items (with loadings greater than 0.3)—along with two Emotional Response items and two Social Interaction items; accounting for 9% of the variance. The fourth factor included five of the eight Emotional Response items (loadings greater than 0.3) along with one Social Interaction item that accounted for 6% of the variance.

In all, 34 of the 44 items loaded on the same subscales were included in GARS-3 and only one item from the Emotional Response subscale (extreme reaction to loud noises) did not load on any factor. Moreover, the decreasing amount of variance across the four factors mirrored that reported for the GARS-3 US sample.

This analysis was repeated with children who had a diagnosis of ASD (as they had formed the sample in the validation of GARS-3) although the sample here was much smaller (*n* = 388). In total, 38.6% of the variance was accounted for by the four factors, but the item loadings were almost identical to those described above.

A factor analysis was also undertaken of the ratings given for the 14 additional items by children who were verbal (*n* = 427) on the two subscales included in GARS-3. The two factors were confirmed with six of the seven Cognitive Style items loading on one factor and five of the seven Maladaptive Speech items on a second factor (loadings greater than 0.5).

A further analysis was then undertaken to confirm a six factor solution with verbal children using a Varimax rotation that was found to account for 43% of the variance. The first factor largely replicated the Social Communication items of GARS-3 with eight items (out of nine) loading greater than 0.5 and accounting for 10% of the variance. The second factor consisted of seven Social Interaction plus two Behaviour items and one Emotional Response item, accounting for 8.6% of the variance. The third factor consisted largely of Behaviour items (8 in all) but also included three Social Interaction items; three Maladaptive Speech items and one Emotional Response item, together accounting for 8.5% of the variance. The fourth factor consisted of six of the seven Cognitive Style items with 6.7% of the variance accounted for and the fifth factor had five of the eight Emotional Response items accounting for 4.9% of the variance. The sixth factor was the least well-defined and consisted of three Behavioural items, two from Social Interaction and one from Maladaptive Speech, accounting for 4.7% of the variance.

In all, 35 of the 56 items loaded distinctly on the same subscales as the GARS-3, although all but one item (from the Emotional Response scale as noted earlier) loaded on one of the six factors. However, the Maladaptive Speech factor was not well defined in this sample of verbal children, as these items tended to be associated with other factors.

### 3.2. Subscale Scores

As the factor analyses had broadly confirmed the suitability of the six factors when used with the sample of children from Kurdistan; further analyses were then undertaken into the reliability of the subscales.

#### Internal Reliability

A raw score was computed for each child on the six subscales. Table 1 reports the internal consistency of the subscales using Cronbach’s Alpha for both Kurdish and American samples.

These reliabilities were highest for the total scale scores for both the four and six subscales, and also high for the Social Interaction, Social Communication and Behaviour factors; all of which were similar to those reported for the U.S. sample. The internal reliabilities were lower for Cognitive Style, Maladaptive Speech and Emotional Responses, which reflects the outcomes from the factor analysis. The Emotional Response and Cognitive Style subscales are less internally consistent than was reported for the U.S. sample, as is Maladaptive Speech in both samples, which is suggestive of the greater individual variation of these factors.

Table 2 presents the inter-correlations of the six subscale scores with the summated score for children who used verbal communication.

The subscales that correlated highest with the total scores were Social Interaction, Restricted/Repetitive Behaviours and Maladaptive Speech. However, the inter-correlations among the other subscales were more modest, although still statistically significant (*p* < 0.001). Again, this confirms the findings from the factor analysis.

When the correlations are calculated across the four subscales on which all children were rated, again it was scores on Social Interaction (r = 0.892), on Behaviour (r = 0.860) and on the Social Communication (r = 0.818) subscales that correlated highest with the total score, with Emotional Responses having a smaller correlation (r = 0.735). The inter-correlations among the four subscales were lower again.

### 3.3. Test-Retest Reliability

A sample of children were retested on GARS-3 some two weeks after the initial assessment. Scores were calculated for the six subscales along with total scores on four and six subscales, and Pearson Product Moment correlations were calculated between scores on first and second ratings. Table 3 represents the correlations between the subscales scores and the total scores at both time points.

These correlations were highly significant (*p* < 0.001) and higher than those reported for the U.S. sample, which ranged from 0.76 to 0.87. Furthermore, there were no statistically significant differences in the scores on the two administrations of GARS-3 (paired *t*-test *p* > 0.05).

### 3.4. Inter-Group Differences

In order to determine if the GARS-3 scores discriminated among the four groups, an Analysis of Variance was undertaken of the mean scores for the four subgroups. As Table 4 shows, there were significant statistical differences (*p* < 0.001) across the four groups on the mean total scores for both the four and six subscales.

Also the range of scores as shown by the standard deviations (SD) was particularly high for the children with ASD.

Tukey’s post hoc tests were used to compare each of four groups with one another. These showed that the total scores for children with ASD were significantly higher than the other three groups (*p* < 0.001). However, the differences between the means scores for children with an ID and with communication language disorders were not significantly different, but both of these groups had significantly higher scores than the mean scores of the typically developing children (*p* < 0.001).

### 3.5. R.O.C. Analysis

An ROC analysis was undertaken to determine the sensitivity and specificity of using the total scores to distinguish between children with ASD from typically developing children. A second analysis examined the distinction between children with ASD from those with intellectual disabilities or communication disorders.

Table 5 summarises the Area Below the Curve statistics derived from an R.O.C. curve analysis along with 95% confidence intervals (C.I.s). The closer this is to 1.00 the better the discrimination obtained on GARS-3 total scores across both the four subscales (including non-verbal children) and the six subscales (for verbal children).

The discrimination was better for the comparison between AS and typically developing children, and less so but still reasonable between AS and those with other developmental difficulties.

Using these analyses, it was possible to identify a cut-off point on the total scale scores that best discriminated the children with ASD from those of typically developing children. For the total score on four subscales, a score of 12 and above proved best at separating the two groups, with a sensitivity of 0.992 and specificity of 0.880. For the present sample, using this cut-off, it would have resulted in eight children in the AS group (2.1%—false negatives) NOT scoring above the threshold and 10 typically developing children scoring above the cut-off (7.5%—false positives). On the six-subscale total, this resulted in a sensitivity of 0.994 and specificity of 0.946. For the present sample, using this cut-off, it would have resulted in one child in the AS group (0.6%—a false negative) NOT scoring above the threshold and seven typically developing children scoring above the cut-off (5.4%—false positives). Although the differences are marginal between the two total scores, the addition of the verbal items does seem to improve the specificity of the scoring.

However, a similar R.O.C. analysis showed that GARS-3 was not as good in discriminating children with ASD from children with I.D. and communication disorders. For the totals on the four subscales, a score of 25 and above proved best at separating the two groups with the sensitivity of 0.912 and specificity of 0.702. For the present sample, using this cut-off, it would have resulted in 60 children in the ASD group (15%—false negatives) not scoring above the threshold, and 40 children with I.D. or communication disorders above the cut-off (18%—false positives). On the six-subscale total, this resulted in a sensitivity of 0.965 and a specificity of 0.626. For the present sample, using this cut-off, it would have resulted in six children in the ASD group (3.5%—a false negative) NOT scoring above the threshold, but 49 children with I.D. or communication disorders scoring above the cut-off (37%-false positives). Hence, using the total score of six subscales in particular will result in possibly many false positives for ASD among children with I.D. or communication disorders, albeit that the specificity was better than when using the four subscale totals. This mirrors the findings with the U.S. samples [8].

### 3.6. Standardised Scores

GARS-3 provides an opportunity to standardise children’s scores by comparing them with the normative data available from the U.S. sample of children with ASD. Although a normative sample of Kurdish children would be preferable, this approach could be tested in the present study.

An autism index was calculated based on the scores on the four or six subscales. The children could then be classed either as unlikely to have ASD or having the possibility of having ASD This grouping could then be cross-tabulated with similar groupings based on the cut-offs identified on the total scores, as described earlier. This showed that there was a 95% concordance on the grouping for children with ASD; an 88% correspondence for those with ID; a 92% concordance for children with communication disorders, and 85% concordance for typically developing children.

A more detailed examination showed that for children with ID, the autism index grouping was more likely to assign them as likely to have ASD than did the cut-off score. For the TD children, the cut-off scores suggested that more would fall into the ASD grouping than the autism index grouping. However, the numbers involved in these analyses were small given the high level of concordance even with these children.

The autism index also provides an indication of the level of severity of the child’s autism. To explore the relationship between these and the totals based on the raw scores, the mean scores for children in each level were calculated as shown in Table 6, and an Analysis of Variance confirmed that the differences were statistically significant (*p* < 0.001), with large effect sizes.

Although the standard deviations are large, there is little overlap in the scores of children across the groupings, which suggests that there could be value in using the U.S. norms to provide a measure of the level of severity for children who are identified as likely to have ASD.

## 4. Discussion

This study was unique in a number of ways. It is apparently the first to examine the psychometric properties of a translated version of a tool developed in the United States when used in another culture and country. This step is often omitted when measures developed in one country are applied to different cultures and other languages. This exercise not only confirms the applicability of the scale for local use but underscores the possible universality of the concepts on which the scale is built.

### 4.1. Process of Translation

The first step in the study was to design and replicate the process for undertaking a psychometric evaluation of GARS-3. The procedures are well-established in terms of translation safeguards, consultation with local experts, and pilot testing with a range of informants and assessors, as described earlier. Such steps are not demanding in terms of resources.

The next step—determining the psychometric properties—is essentially a replication of the procedures used by the test designers, and this can be more demanding in terms of gaining access to sufficient numbers and types of participants, as well as having the personnel trained and available to administer the scale. Fortuitously, thorough procedures had been used in developing a third revision of the scale, which may not always be the case. Hence, in selecting measures for use in other cultures, careful attention should be paid to the psychometric properties of the original scale. If these are poor, then there is a good chance that this will also be the case with any translation, although some adaptations might improve the psychometric properties.

The study provides an example of how the psychometric properties were assessed in a culture and country with relatively few resources and traditions in undertaking research and evaluation. The study was led by the first author who had extensive experience in Iran of assessing the suitability of various autism assessment and diagnostic tools [8,13]. His role was crucial to instigating and guiding the process.

A second advantage was locating the study within a well-established center for children with developmental disabilities in the capital city. Existing personnel were available to administer the scale and also a pool of potential participants was available through past or current children and families known to the Center. In addition, existing networks could be enlisted to widen the recruitment. Basing the study within an existing service provides opportunities to offer ongoing support to families whose children are identified as likely to have a developmental disability such as ASD. This reduces the ethical dilemma of identifying needs without making arrangements for follow-up that sadly can be common, even in well-resourced countries [14].

A third requirement was fulfilled in the study, namely the training provided to the assessors and the checks that were made to ensure that they followed the protocol for administration so that consistency was maintained across different assessors. This step also had the side-effect of encouraging teamwork among staff from different disciplines around a common tool and for designing multi-disciplinary interventions to support the families and the children’s development [15].

The final step involved the combining of information from individual paper records into a computer database to enable statistical analyses to be undertaken. The first and last authors were well experienced in undertaking these tasks, but such expertise may not be readily available in less affluent countries. That said, there may be statisticians available in local universities or government departments whose help can be enlisted, or there may be connections made with personnel in other countries to assist with data analyzing and report writing, as happened here.

The foregoing summary relates to the first aim of the study and is offered as a model that others may follow, as it has been replicated by ourselves in relation to different ASD tools, as well as by others [11,16].

### 4.2. The Suitability of GARS-3 in Kurdistan

Turning to the second aim of the study, the findings broadly confirm the use of GARS-3 as an assessment tool for ASD in Kurdistan, in particular and also for neighboring countries with a shared culture and language. The factor structure of the Kurdish translation largely replicated that of the U.S. version, especially in terms of the three main factors of Social Interaction, Social Communication and Repetitive/Restrictive Behaviours, which are core indicators for ASD as defined in DSM-5. In addition, the internal reliabilities of these subscales, their correlations with total scores and the test-retest reliabilities suggests that they are robust measures. The other three subscales, and especially those based on verbal communication, show greater disparity from the U.S. analyses. In part this could reflect cultural variations that studies have reported that involved certain items on other assessment tools for ASD [17,18]. Nonetheless, there could still be value in using the items from the other subscales, especially with children who use verbal communication.

The predictive validity of the GARS-3 was assessed by examining the differences in summary scores across the four groups of children. Children with ASD had significantly higher scores than typically developing children. Cut-off scores were identified that yielded high sensitivity and specificity in determining children more likely to have ASD, with only small proportions of false negatives and false positives. Furthermore, there was a striking correspondence between the autism index measure based on U.S. norms and the groupings based on the cut-offs derived from the Kurdistan data, all of which is suggestive of strong psychometric properties.

However, the total scale scores are more reliable and valid than subscale scores in both the U.S. and Kurdistan versions. Caution is therefore needed in making comparisons among the various subscales, although they may give some insights into the strengths and weaknesses of certain children. Indeed, the wide variation in scores found among children with ASD in the study is reflective of the condition and the need for individual focused interventions [18,19]. Moreover, the overall amount of variance explained in this study and that in the U.S. was less than 50%. This variance indicates that children with ASD tended to show distinctive and expected behaviour patterns across the item set.

GARS-3 was less successful in distinguishing children with ASD from those who had other developmental disabilities, particularly intellectual disability and communication disorders. In one sense this is not surprising, as the items describe behaviours that are also indicative of these other conditions [20]. There are indications from the analyses undertaken that higher scores on the GARS-3 are needed to distinguish children with ASD from other disabilities, and in any case, higher scores are most apparent with more severe forms of ASD. However, this runs the risk of children with milder forms of ASD being returned as false negatives. Conversely, there is the possibility of co-morbidity of ASD with other developmental disabilities that may also account for the poorer differentiation. Therefore, children with I.D. and communication disorders may also score above the lower threshold for milder forms, and further assessments should be sought to confirm the presence of autistic traits [20].

It must be emphasized that second level screening tools such as different versions of the GARS scale should not be applied to make a confirmed diagnosis of ASD, and its application needs to be triangulated through the cooperation of multi-disciplinary evaluations for individuals who screened at risk based on it and also for those who may have other types of developmental impairments who need additional evaluations [3]. The use of ‘gold standard’ assessments such as ADI-R and ADOS is also recommended, especially as their relevance to other cultures has been confirmed [13,21].

### 4.3. Limitations

The findings of this study should be considered with respect to its limitations. Caregivers of individuals with ASD had already received a diagnosis, hence their judgments about the scale’s items may have been affected by knowledge of the significance of their child’s behaviours, which they may not have had before receiving a diagnosis. A prospective study with a suspected sample of those who may have ASD would additionally establish the utility of GARS as a screening tool either in its complete form or in a shorter version by means of some adopted items described in a related paper [22]. This investigation would comprise diagnostic tools to use with samples of individuals who screen both negative and positive.

Another limitation in the present study was that there was a confirmed diagnosis of ASD or intellectual disability for the individuals who participated (some of whom may also have ASD). Although the confirmation of the final diagnosis is a desirable factor, this would be difficult to attain in an area such as the Kurdistan region of Iraq given the scarcity of trained professionals.

### 4.4. Further Research

These findings also have international application in that they confirm the universality of certain social communication and behavioral indicators of autism in children which could be further tested by using the GARS in other cultures. Previous studies using an earlier version of GARS in Iran [5] and Turkey [7] would suggest that GARS-3 could prove suitable in other middle-eastern countries.

Further prospective research is needed to confirm the assessment diagnosis with children who score above and below the cut-off scores identified in this study. Moreover, the development of normative data drawn from Kurdish samples of children would be advantageous, although ambitious, given the lack of diagnostic services in many low and middle-income countries.

However, studies that have directly compared children across countries have found some differences in the extent to which items relating to socialisation, verbal communication, and restricted interests were reported, although all were present across the countries [23,24]. This suggests that cultural variation exists in the perception of ASD-related traits that reflects the parental values or perceptions regarding their children’s behaviors as being labeled unusual within their particular culture. There may be further and/or different items that should be added to the translated tools to improve their reliability and validity. Future research could assist with the development of culturally sensitive screening and diagnostic tools [2]. This may also assist in preventing the later and in reducing reports of ASD identification among ethnical minority groups who immigrate to Western countries [25,26].

## 5. Conclusions

A process was described for confirming the suitability of assessment tools for ASD for use in other countries that utilized existing resources. The factor structure of GARS-3 was broadly replicated, especially on items relating to social interaction, social communication, and behaviours. The cut-offs for the total scores on the measure that were indicative of possible ASD had a high degree of specificity and sensitivity in distinguishing children with ASD from their typically developing peers. However, some children with I.D. and communication disorders may also score above the threshold, and further assessments should be sought to confirm the presence of autistic traits.

Although GARS-3 could be recommended for use in Kurdistan and possibly similar cultures, further prospective research is needed to confirm a diagnosis of assessment with children who score above and below the cut-offs-identified in this study. Moreover, the development of normative data drawn from Kurdish samples of children would be advantageous, although ambitious given the lack of diagnostic services in many low- and middle-income countries. Nonetheless, over time, sizeable samples could be accumulated, especially if data were pooled across different providers of assessment and diagnostic services with suitable safeguards for confidentiality and consent were utilized.

## Figures and Tables

**Table 1:** The subscales and total items on GARS-3 for the Kurdish and the U.S. samples and the Cronbach Alpha coefficients amount.

Scales	Alpha (the Kurdish Sample)	Alpha (the American Sample)
Total score 4 subscale	0.944	0.94
Total score 6 subscales	0.936	0.93
Social Interaction	0.901	0.94
Social Communication	0.903	0.89
Restricted/repetitive behaviours	0.841	0.90
Emotional responses	0.733	0.90
Cognitive style	0.637	0.86
Maladaptive speech	0.700	0.79

**Table 2:** The raw scores on the six subscales and total score across 56 items and their Pearson product moment correlations (*n* = 427).

	Social Interaction	Social Communication	Emotional Responses	Cognitive Style	Mal-Adaptive Speech	Total Six Scales
Restricted/Repetitive Behaviour	0.739	0.551	0.585	0.500	0.627	0.814
Social Interaction		0.590	0.550	0.386	0.653	0.845
Social Communication			0.485	0.458	0.545	0.808
Emotional Responses				0.469	0.450	0.712
Cognitive Style					0.514	0.651
Maladaptive Speech						0.794

**Table 3:** The test-retest ratings on the three subscales with summary statistics at each time point and their Pearson product moment correlations (*n* = 37).

	r	Mean (SD) Time 1	Mean (SD) Time 2
Restricted/repetitive behaviours	0.993	2.76 (4.69)	2.87 (4.72)
Social Interaction	0.995	4.21 (7.23)	3.90 (6.50)
Social Communication	0.996	5.76 (7.10)	5.83 (7.11)
Emotional responses	0.988	5.07 (4.93)	5.05 (4.78)
Cognitive style	0.996	2.95 (3.18)	2.86 (3.11)
Maladaptive speech	0.997	1.05 (2.13)	1.08 (2.17)
Total score four subscales	0.996	13.00 (13.47)	13.19 (13.75)
Total score six subscales	0.998	17.11 (18.09)	17.02 (17.84)

**Table 4:** The means (SD) and range for the GARS-3 total scores across the four groups of children.

Scales	ASD *n* = 388	Intellectual Disability *n* = 165	Communication Disorders *n* = 49	Typically Developing *n* = 133	F
Total score 4 subscales—(min 0—max 132)	45.67	18.35	15.57	4.86	259.3
Mean (Standard deviation)	(19.84)	(14.71)	(9.31)	(7.33)	(*p* < 0.001)
Total score 6 subscales—(Min 0: max 174)	46.32	21.88	17.70	5.64	219.4
Mean (Standard deviation)	(17.78)	(13.70)	(8.47)	(8.05)	(*p* < 0.001)

**Table 5:** Results from R.O.C. Analysis.

	ASD v Normal Development	ASD v ID+ Communication Disorders
Total score four subscales	0.986 (95%CI 0.975–0.997)	0.896 (95%CI 0.868–0.924)
Total score six subscales	0.989 (95%CI 0.978–1.000)	0.896 (95%CI 0.860–0.933)

**Table 6:** Means (and SD) on total scores grouped by level of disability using autism index from GARS-3.

	Not ASD	Level 1 Support Required	Level 2 Substantial Support Required	Level 3 Very Substantial Support Required
Nonverbal Children	6.00 (3.06)	20.57 (8.08)	55.65 (13.90)	90.93 (5.93)
(4 subscales)	(*n* = 17)	(*n* = 92)	(*n* = 178)	(*n* = 14)
Verbal Children	3.04 (3.96)	23.10 (9.22)	54.86 (14.09)	No Variation
(6 subscales)	(*n* = 119)	(*n* = 194)	(*n* = 115)	(*n* = 1)
